# Effectiveness of internet-supported cognitive behavioral and chronobiological interventions and effect moderation by insomnia subtype: study protocol of a randomized controlled trial

**DOI:** 10.1186/s13063-015-0790-2

**Published:** 2015-07-04

**Authors:** Kim Dekker, Jeroen S. Benjamins, Annemieke Van Straten, Winni F. Hofman, Eus J. W. Van Someren

**Affiliations:** Netherlands Institute for Neuroscience, An Institute of the Royal Netherlands Academy of Arts and Sciences, Meibergdreef 47, 1105 BA Amsterdam, The Netherlands; Department of Clinical and Health Psychology, Department of Experimental Psychology, Utrecht University, Heidelberglaan 1, 3584 CS Utrecht, The Netherlands; Department of Clinical Psychology, VU University Amsterdam & EMGO Institute for Health Care and Research, Van der Boechorststraat 1, 1081 BT Amsterdam, The Netherlands; Department of Psychology, Brain and Cognition group, University of Amsterdamy, Weesperplein 4, 1018 XA Amsterdam, The Netherlands; Personal Health Institute International, Lobo-Braakensiekstraat 94, 1065 HP Amsterdam, The Netherlands; Departments of Integrative Neurophysiology and Medical Psychology, Center for Neurogenomics and Cognitive Research (CNCR), Neuroscience Campus Amsterdam, VU University and Medical Center, De Boelelaan 1085, 1081 HV Amsterdam, The Netherlands

**Keywords:** insomnia, chronobiological treatment, cognitive behavioral therapy, internet, actigraphy, personalized medicine, stratified medicine

## Abstract

**Background:**

DSM-V criteria for insomnia disorder are met by 6 to 10 % of the adult population. Insomnia has severe consequences for health and society. One of the most common treatments provided by primary caregivers is pharmacological treatment, which is far from optimal and has not been recommended since a 2005 consensus report of the National Institutes of Health. The recommended treatment is Cognitive Behavioral Therapy for Insomnia. Effectiveness, however, is still limited. Only a few studies have evaluated the effectiveness of chronobiological treatments, including the timed application of bright light, physical activity and body warming. Another opportunity for optimization of treatment is based on the idea that the people suffering from insomnia most likely represent a heterogeneous mix of subtypes, with different underlying causes and expected treatment responses.

The present study aims to evaluate the possibility for optimizing insomnia treatment along the principles of personalized and stratified medicine. It evaluates the following:

1. The relative effectiveness of internet-supported cognitive behavioral therapy, bright light, physical activity and body warming;
2. Whether the effectiveness of internet-supported cognitive behavioral therapy for insomnia can be augmented by simultaneous or prior application of bright light, physical activity and body warming; and
3. Whether the effectiveness of the interventions and their combination are moderated by the insomnia subtype.

**Methods/Design:**

In a repeated measures, placebo-controlled, randomized clinical trial that included 160 people diagnosed with insomnia disorder, we are evaluating the relative effectiveness of 4 intervention weeks. Primary outcome is subjective sleep efficiency, quantified using a sleep diary. Secondary outcomes include other complaints of sleep and daytime functioning, health-related cost estimates and actigraphic objective sleep estimates. Compliance will be monitored both subjectively and objectively using activity, light and temperature sensors. Insomnia subtypes will be assessed using questionnaires. Mixed effect models will be used to evaluate intervention effects and moderation by insomnia subtype ratings.

**Discussion:**

The current study addresses multiple opportunities to optimize and personalize treatment of insomnia disorder.

**Trial registration:**

Netherlands National Trial Register NTR4010, 4 June 2013.

## Background

Dissatisfaction with sleep is reported by about 25 % of the adult Western population [1]. The prevalence is even higher in older people and twice as high in women as in men [2, 3]. When complaints about difficulties falling asleep, maintaining sleep, early morning awakenings or nonrestorative sleep are accompanied by physical or cognitive daytime complaints, and last for at least 3 months, occurring at least three times a week, without being secondary to environmental factors or comorbidities that prohibit sleep, a person suffers from insomnia disorder, according to the DSM-V [4]. These criteria are met by 6 to 10 % of adults [2]. Daytime complaints of people with insomnia concern cognitive functioning [5], depressed mood [6] and fatigue [6]. Additionally, people reporting insomnia or low sleep quality have higher risks of depression [7], metabolic diseases and cardiovascular problems [8]. These daytime consequences and comorbidities lead to a reduction in work productivity, to increased sick leave and healthcare consumption and consequently to high economic costs. In summary, insomnia has severe consequences for health and society [9], underscoring the importance of optimizing interventions to promote better sleep. To do so, the regulation of sleep needs to be considered.

### Circadian rhythm and sleep

Virtually all behavioral and physiological processes show a 24-h rhythm even in the absence of environmental time cues. This circadian (from circa = about, and dies = day) rhythm is, for example, present in sleep and wakefulness, hormone secretion, alertness, body temperature, metabolism, perception and cognition. Endogenous clock mechanisms that promote the expression of 24-h rhythms are located in all human cells [10] and are synchronized by the central clock of the brain, the hypothalamic suprachiasmatic nuclei (SCN). The SCN is considered the major clock because only SCN tissue is able to maintain a circadian rhythm in the absence of external input [10]. Under normal circumstances, several exogenous cues, or zeitgebers, entrain the SCN. Light is the strongest zeitgeber [11]. The retinohypothalamic tract (RHT) is one of the three major input pathways of the SCN, providing it with information from photoreceptor cells in the retina. Secondary zeitgebers include, for example, the temperature of the environment, body core and skin; metabolism; and the rest-activity cycle. When the circadian rhythms of the body are synchronized with the zeitgebers, the rhythm is optimally entrained. A type of insomnia that is characterized mostly by complaints about initiating sleep may be due to a misalignment of the circadian rhythm in sleep-promoting physiology and the sleep window the patient tries to adhere to, typically between 23:00 and 7:00 [12, 13].

### Homeostasis of sleep

In addition to the circadian regulation of sleep and wake, a homeostatic component affects sleep propensity. Whereas the circadian process is relatively independent of prior sleep or wake, the homeostatic process on the other hand is sensitive to the sleep-wake history [14]. The sleep homeostatic process increases sleep propensity during wakefulness until sleep is initiated. Sleep propensity declines during sleep. The biological mechanisms underlying sleep homeostasis are less well characterized than the mechanisms underlying circadian regulation, but likely involve astrocyte-dependent adenosine accumulation [15, 16].

Some types of insomnia may involve altered homeostatic regulation of sleep propensity [17].

### Sleep-permissive and wake-promoting conditions

Although the regulation of sleep and wakefulness has been well modeled by interacting circadian and homeostatic processes in lab studies, additional sleep-permissive and wake-promoting conditions have been largely overlooked [18]. Most people sleep best lying in bed, with the lights off and the curtains drawn, in a thermally agreeable, safe and relatively quiet environment. These parameters (posture, temperature, environmental light and sound, fear) moderate the expression of sleep and wake. Whether altered sensitivity to sleep-permissive and wake-promoting conditions might be involved in the sleep complaints of some types of insomnia remains virtually unexplored [19–21].

### Treatment of insomnia

Even though the underlying causes of insomnia are at present insufficiently understood, one of the most common treatments provided by primary caregivers (mainly general practitioners) is sedative or hypnotic drugs mostly benzodiazepines or benzodiazepine receptor antagonists [1]. Unfortunately, pharmacological treatments have a high prevalence of adverse effects. Daytime drowsiness, risk of abuse and addiction, and rebound insomnia on withdrawal are a few examples of these side effects [22]. Pharmacological treatment is therefore not recommended [23].

In the past decades, two other interventions have been developed. The first intervention is cognitive behavioral therapy for insomnia (CBT-I). It has similar or even better and more prolonged outcomes than pharmacological treatment, and lacks the negative side effects [24]. A second, less evolved, but promising type of intervention is chronobiological treatment (CT). Chronobiological treatments aim to enhance regular and appropriately timed input to the circadian timing system to support its role in sleep regulation, by use of bright light, body warming or physical activity (for example, [21, 25, 26], for a review see [27]). These manipulations may become more important with increasing age, to counteract age-related changes in the functional neuroanatomy of the biological clock of the brain [28].

### Cognitive behavioral therapy

Cognitive behavioral therapy for insomnia (CBT-I) is a combination of cognitive and behavioral treatments. CBT-I usually includes stimulus control (that is, association of bed with sleeping), sleep restriction (that is, restrict an individual’s time in bed to average sleep time), relaxation training, cognitive therapy (that is, diminish misconceptions about sleep) and sleep hygiene (for example, general guidelines about behavioral and environmental factors that influence sleep) [29]. CBT-I has been shown to be effective in numerous randomized clinical trials [24, 29–34]. A problem facing implementation on a large scale is the lack of skilled therapists.

### Chronobiological treatment

Three chronobiological manipulations have been applied successfully and will be discussed below.

#### Bright Light (BL)

Bright light, including natural daylight, entrains the circadian rhythm [35]. Retinal ganglion cells projecting to the SCN through the retinohypothalamic tract (RHT) inform the circadian timing system about the intensity of light exposure. The SCN uses this information to entrain its intrinsic rhythmic activity to the 24-h light–dark cycle [10]. Studies in humans show that phase shifts can be imposed by manipulation of the light–dark cycle [35, 36]. A phase advance can be induced by means of bright light given in the morning, especially closely after the body core temperature minimum; a phase delay can be induced with light applied in the interval from late afternoon to just prior to the body core temperature maximum [35, 37, 38]. The effect of light on sleep may not be limited to changing the sleep phase, as suggested by studies that found enhanced sleep quality in the absence of clear rhythm shifts [25, 39].

#### Body and skin warming (BW)

Core body temperature shows a clear 24-h rhythm. In normal sleepers with a sleep period between 23:00 and 7:00, the temperature peaks between 18:00 and 20:00 h and has its minimum between 4:00 and 5:00 h [11]. Apparently, the connection between sleep or vigilance and core body temperature is strong. We sleep when core body temperature is low and are awake when it is high [18]. In contrast, skin temperature is increased during the sleep period because of increased skin blood flow in response to a supine posture [40]. Increased skin blood flow promotes heat loss, resulting in a lower core body temperature. Romeijn *et al*. [18] reviewed the effect of manipulation of skin temperature on vigilance, and they explain in more detail the underlying processes. Several studies that applied water-perfused thermo suits to increase skin temperature slightly within the thermoneutral zone showed that this manipulation enhances sleep propensity [18]. This effect has not only been demonstrated in healthy adults, but also in people with insomnia [20, 21]. A more practical and home-applicable version of this temperature manipulation is to make use of the body’s thermoregulatory mechanisms and warm the body prior to sleep by mild physical activity or taking a hot bath [41]. About two hours later, core body temperature has returned to baseline, while skin temperature is still somewhat elevated. This pre-sleep body warming approach has been used with success in insomnia [42].

#### Physical Activity (PA)

As diurnal mammals, humans are active during the day and sleep at night. The rest-activity profile of people with a very inactive lifestyle, for example sedentary elderly, shows a small 24-h amplitude. Several studies evaluated the effect of enhancing physical activity or exercising on this amplitude and on sleep [26, 43–51]. Two possible mechanisms have been proposed to be involved in the effect of enhanced physical activity on sleep. The thermoregulatory hypothesis proposes that exercise exerts an effect on sleep through initially increasing core body temperature. This subsequently triggers heat loss to decrease the temperature, which is conducive to sleep. The body restoration hypothesis proposes that exercise depletes energy levels and promotes sleep because it increases the need to activate restorative mechanisms. In 2000, Driver & Taylor reviewed the studies done up until that year [46]. Unfortunately, most studies use good sleepers. Their conclusion was that exercise of moderate intensity and focusing on endurance rather than peak intensity, was most beneficial for perceived sleep quality. Tanaka *et al*. [51] showed that exercise is most effective around 17:00 h, supporting the thermoregulatory hypothesis. Benloucif *et al*. [43] however found equal effects of morning and evening exercise on subjective sleep quality. More recently, Passos *et al*. [50] summarized studies using exercise in primary insomnia. This review demonstrated improvement in sleep quality. Additionally, they discuss possible explanations for the acute and chronic effects of physical activity on primary insomnia [50].

### Optimizing treatment and accessibility

Whereas the combination of CT and CBT-I may be more effective than their use in isolation, and has successfully been applied in only one study [30, 52], no prior study addressed the relative contribution of the different interventions. More research is therefore needed to compare effectiveness of different combinations of therapies; one of the aims of the present study.

A problem facing implementation of CBT-I is the shortage of skilled therapists. Internet-based CBT-I may alleviate this problem [53]. Internet-based therapy is highly self-guided, structured and personalized. Knowledge-based technology renders a personalized concept consult, which can be adjusted by therapist for situations that cannot be pre-programmed. This saves a lot of time for a therapist and subsequently may help to solve the discrepancy between care needed and care available [33, 34], thus allowing for large-scale implementation.

Another opportunity for the optimization of treatment is based on the idea that the people suffering from insomnia most likely represent a heterogeneous mix of subtypes, with different underlying causes and expected treatment response. These subtypes may not only be distinguished by type of sleep problems. Other factors such as personality traits, medical complaints and medical history, the ability to perceive comfort [21, 54–56] may represent variables that differ between the proposed subtypes as well. Our research group commenced a large-scale study using web-based assessment of questionnaires to collect data on these variables (the Netherlands Sleep Registry, NSR, www.sleepregistry.org). The use of latent class analysis on these data will allow for the data-driven detection of multivariate subtypes, calculation of subtype probability for participants according to this data-driven subtype nosology, and evaluation of differential treatment responses, depending on the subtype probability.

The present study aims to evaluate the possibility to optimize insomnia treatment along the principles of personalized and stratified medicine, by making the first step towards the development of a protocol for individualized optimal treatment for specific subtypes of insomnia, which each may show different responsiveness to CBT-I and the different CT manipulations.

### Objectives

The objectives of this study are as follows:

1. To evaluate the relative effectiveness of internet-based CBT-I, three different types of CT (light, temperature and physical activity), and the combined application of CBT-I and each of the CT’s, as compared to placebo treatment, on [1] subjective sleep efficiency (primary outcome), [2] subjective daytime functioning, [3] actigraphic sleep estimates, and [4] cost effectiveness.

2. To evaluate whether insomnia subtype moderates the effectiveness of the individual and the combined treatments.

3. To evaluate whether the effect of CBT-I is enhanced by prior CT.

## Methods/Design

### Trial design

**Fig. 1:**
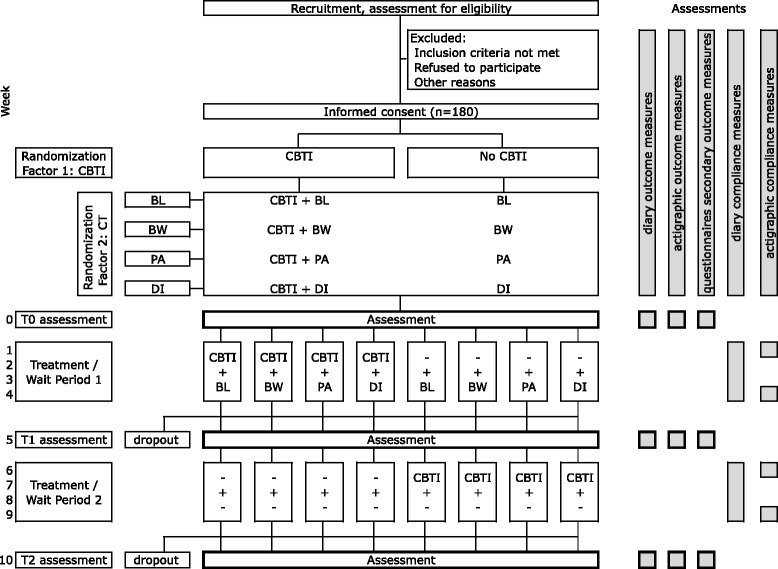
Flow chart of the trial design and outcome assessments. In the recruitment phase, participants fill out a questionnaire to assess their eligibility to participate. This questionnaire includes the Duke Structured Interview for Sleep Disorder Diagnosis (30 min). During weeks 0, 5 and 10, participants fill out questionnaires and sleep diaries and will wear an actigraph for subjective and objective sleep estimates, respectively. The diary is continued in all other weeks to support cognitive behavioral therapy (CBT). During weeks 1, 4, 6 and 9, participants will wear actigraphs for objective measures of compliance to chronobiological treatment

The study is a double blind, randomized, placebo controlled trial. Aiming at 160 participants who complete the trial, with a dropout rate of 10 %, 180 participants will be recruited, who will be randomly assigned to one of eight limbs in a repeated measures design with two factors (CBT-I and CT), of respectively 2 levels (active CBT-I treatment versus waitlist monitoring) and 4 levels (light, physical activity, body warming and placebo) (see Fig. 1). Covariate-adaptive randomization aims at an equal representation of insomnia subtypes within each treatment limb. Assessment will be performed at baseline (week 0), after a 4-week treatment period (week 5), and after a second 4-week treatment/monitoring period (week 10).

### Participants

#### Inclusion and exclusion criteria

Participants will be included that meet the criteria of an insomnia disorder as defined in the DSM-V. Furthermore, participants have to be between 18 and 70 years old. Because a meta-analysis [57] indicated that insomnia is more prevalent in females (odds ratio (OR) = 1.41), we aim for inclusion of 60 % females and 40 % males.

This study follows the Helsinki Declaration’s principles, meaning that all patients sign a written informed consent stating that participation is voluntary and that participation can be withdrawn at any time, without any negative consequences concerning their current or future treatment. Approval was obtained on 21 May 2013 from the medical ethical committee of the VU University medical center in The Netherlands (Protocol 12/472). The trial is registered at the Netherlands National Trial Register (NTR4010) [58].

Participants are recruited through the Netherlands Sleep Registry and (social) media. Interested candidates fill out a screening questionnaire regarding health status and insomnia diagnostic criteria (relevant items of Duke Structured Interview for Sleep Disorders, DSISD) [59], level of physical activity [60], light exposure (by asking about habitual hours spent outside during daylight hours) and bathing habits (frequency and duration of bathing/showering). Participants will be excluded for the following reasons:

1. their estimated baseline level and timing of activity, bright light or body warming are already similar to the CT interventions planned;
2. they report an eye disease incompatible with light treatment (aphakia or retinopathy), or cardiovascular or movement disorder incompatible with the exercise treatment;
3. they report that they are currently diagnosed with a psychiatric or neurological disorder;
4. they are shift workers, since the treatment protocol does not allow for alternative schedules, and their sleeping problems may not be due solely to insomnia; or
5. they use sleep medication regularly, unless they are willing and able to restrict their usage to a maximum use of twice a week, at least 1 month prior to enrollment.

### Randomization

Participants who meet the criteria and sign informed consent will be randomly assigned to CBT-I or to the waitlist and to one of the four CT conditions (including one placebo). Simple randomization will be applied to the first batch of 48 participants. During the staggered entry of subsequent participants, covariate-adaptive randomization [61] will be applied in order to maintain balanced groups throughout the study. The covariates are age, sex, use of non-sleep related medication and time of year. Since some covariates are continuous rather than categorical, randomization will be done following the method proposed by Frane [62]. This method temporarily assigns each new participant one by one to each treatment group and obtains a *P* value of the test for between-group differences for each of the covariates for that assignment, using analysis of variance (ANOVA) for continuous variables and a X^2^ test for categorical variables. This renders a *P* value for each of the four covariates for each of the eight possible assignments. The lowest of the four *P* values thus represents the least balanced covariate in that assignment situation. These lowest, or minimum, *P* values per assignment will then be used to make the actual assignment to one of the treatment conditions, where a participant will be assigned to the group for which this minimum *P* value is highest, thus resulting in a group assignment that keeps imbalance over groups as small as possible. If group sizes become unbalanced (that is, differing by more than five participants), the Frane method will be applied only to the six smallest groups instead of all eight. All randomization will be done using R [63]. Randomization will be scripted, so actual group assignment is automated.

### Blinding and expectation assessment

A patient information letter explains the four CT manipulations (including placebo CT) and CBT-I). All treatments in the study are presented to the participants as possibly effective. It is not possible to blind participants to the different treatment conditions. Since all information is given via email or through the postal service, there will not be a blinding of instructors. However, CBT-I counselors are instructed not to correspond about the expected outcome of CT treatments, and participants will be asked not to mention their CT condition in possible correspondence with the CBT-I counselor. When participants, however, do reveal their condition, the CBT-I counselor will be replaced. In order to secure blinded data analysis, information regarding treatment conditions will be coded. Only once data processing is finished and the dataset is finalized, will the code will be broken. The analyst (KD) will not have access to the key document. Because all outcome assessments are either self-reported through the internet or obtained from recording devices, blinding of the assessors in not applicable. Judgments of the participant on expected effectiveness will be assessed at T0, T1 and T2 using a 7-point Likert-scale for each of the treatments. After randomization, at T0, participants will be asked about the expectation regarding their assigned CT and CBT-I. After treatment, at T1 and T2, participants will be asked to what extent their sleep problems have changed compared to baseline and to what extent they attribute this change to the treatment.

### Study settings

All measurements and therapies are conducted at the participants’ homes, using the Internet [64, 65]. Treatment devices are sent to their homes. Participants are informed on the types of treatment used in the study and the objective of comparing them.

### Interventions

#### Cognitive behavioral therapy for insomnia

All participants will receive internet-based, personalized cognitive behavioral treatment for insomnia provided through the Somnio website for 4 weeks. The Somnio Internet therapy uses knowledge-based technology to prepare a consult and support the therapist to apply the protocol in a consistent and comprehensive way [66]. The personally assigned CBT-I therapist can adjust each consult if needed. CBT-I will consist of four consults, one every Monday morning. Every morning and evening, participants fill out the Dutch online version of the Consensus Sleep Diary [67]. The first consult will be based on the sleep diary data from the previous week, in combination with the person’s beliefs and attitude towards sleep, as assessed during week 0 by the Dysfunctional Beliefs and Attitudes towards Sleep (DBAS) questionnaire [68]. Sleep diary data of the consecutive weeks will be used to determine which cognitive and behavioral components are emphasized during the treatment [66]. A more detailed description of CBT-I has been provided earlier in this article.

#### Chronobiological treatment

##### Physical Activity

At enrolment, participants are asked to fill out questionnaires to assess their health status as well as the habitual level and timing of activity [60]. The answers on an extended Baecke questionnaire will provide the necessary information to determine the specific personalized implementation if they are randomized to the physical activity condition. More specifically, in the active treatment limb, the most intense physical activity (PA) that participants report to habitually maintain for at least half an hour (for example, (walking, running, cycling) will be (re)scheduled to be performed daily for half an hour preferably starting three hours before ideal bedtime, and never ending closer than two hours prior to ideal bedtime. The physical activity will thus at no point exceed the participants usual duration and intensity, but will be daily and set to a specific time of the day.

##### Body Warming

For body warming (BW), participants randomized to the temperature condition are instructed to take a warm bath daily for half an hour, starting 3 hours before the ideal bedtime and never ending closer than 2 hours prior to ideal bedtime. The physical activity and temperature manipulation procedures will result in elevated skin temperature at bedtime, which can enhance sleep onset [18]. If the manipulations would be done closer to bedtime, core body temperature would not have returned to baseline at bedtime and possibly interfere with sleep [18, 19].

##### Bright Light

Participants randomized to the bright light (BL) condition will receive a Philips goLITE BLU light device (HF3220/01, Philips Consumer Lifestyle, Drachten, The Netherlands). They will be instructed to install the light on a table facing a window to minimize glare by reducing contrast between relatively small bright light source and the background. The light will be set on the side, within the range of vision, but not straight across the participant. This will reduce strain on the eyes. They will be asked to sit facing the light in close proximity for half an hour at a fixed time each morning within an hour after habitual wake-up time, for example during breakfast.

##### Deactivated Ionizer

Many randomized controlled trials testing the effect of morning bright light on seasonal affective disorders (SAD), compare bright light with negative air ionization [69–72]. These studies show that morning High Density Negative Ionization (HDNI) is as effective as morning bright light treatment. Negative air ionization has been shown to positively affect cognitive performance and depression [73]. The application has not been evaluated for its possible effect on insomnia. In one study on SAD, a deactivated ionizer (DI) was used as placebo condition [74]. The ionizer was modified to suggest normal functioning, as indicated by airflow, while negative ion production had been deactivated. Treatment outcome expectancy for the (deactivated) negative ionizer was equal to that of BL. In the present study, participants randomized to the placebo treatment will therefore receive a likewise deactivated ionizer (DI) device (Ionic Air Purifier, XJ-2100, Shanghai Neo.Tec Electron Co., Ltd, Shanghai, China). Participants will be instructed to install the device on a table where they can sit in close proximity to it for half an hour each morning at their earliest convenience after ideal wake-up time, for example during breakfast. This placebo has been applied successfully in several studies in the USA [69, 74], but not yet in the Netherlands, making it unlikely to be recognized as a placebo.

### Assessments and outcomes

To assess subjective sleep parameters as well as daytime complaints, participants will be keeping a diary in the morning and evening for the entire 11-week protocol. Sleep is assessed in the morning using the Dutch version of the Consensus Sleep Diary [67]. Primary outcome, sleep efficiency (the percentage of time slept during the time in bed for sleep), is calculated from the sleep diary variables as follows:

$$ SE\kern0.5em =\kern0.5em \frac{\left( LightOn\kern0.5em -\kern0.5em  LightOff\right)\kern0.5em -\kern0.5em  SOL\kern0.5em -\kern0.5em  WASO\kern0.5em -\kern0.5em EMA}{LightOn-\kern0.5em  LightOff} $$

where *SE* is sleep efficiency; *LightsOn* is the moment people indicated they stop trying to sleep, calculated from their final wake up time and time they stayed in bed to try to fall asleep again; *LightsOff* is the time people turn off the lights and/or disengage from activities to go to sleep; *SOL* is sleep onset latency; *WASO* is the time awake after sleep onset; and *EMA* is early morning awakening, or the time people spend trying to fall back asleep after final awakening.

Daytime functioning is assessed in the evening using a dedicated questionnaire on all complaints listed in the DSM-V and the International Classification of Sleep Disorders, 2nd edition (ICSD-2). During week 0, participants will fill out the complete Duke Structured Interview for Sleep Disorders in order to have a complete overview of sleep complaints and other medical and psychological complaints they currently have or have had in the past.

During weeks 0, 5 and 10, participants will fill out a comprehensive set of questionnaires:

1. Expectations or subjective outcome of the selected treatments;
2. Health-related costs during the last 4 weeks (Trimbos/iMTA questionnaire for Costs associated with Psychiatric Illness: TiC-P) [75];
3. Functional health (Quality of Life Questionnaire, Short Form 36, SF-36) [76];
4. Depression and anxiety (Hospital Anxiety and Depression Scale, HADS) [77];
5. Mood (Positive Affect, Negative Affect Scale, PANAS) [78];
6. Experience of pleasure and comfort (Temporal Experience of Pleasure Scale, TEPS) [79];
7. Thoughts during resting state (Amsterdam Resting State Questionnaire, ARSQ) [55]
8. Hyperarousal. Because this is considered a key feature of insomnia, it is queried using four different scales; the Arousal Predisposition Scale (APS) [80]; the Hyper Arousal Scale (HAS) [81]; the adult ADHD Self-Report Scale (ASRS) [82]; and the Pre-Sleep Arousal Scale (PSAS) [83];
9. Insomnia severity (Insomnia Severity Index, ISI) [84];
10. Beliefs and attitudes regarding sleep (DBAS) [68];
11. Sleep effort (Glasgow Sleep Effort Scale, GSES) [85];
12. Sleep self-efficacy (Sleep Self-Efficacy Scale, SSES) [86];
13. Sleep locus of control (SLOC) [87].

As shown in Fig. 1, ambulatory recordings will be performed during the baseline week and the first week of treatment (weeks 0 and 1), the last week of the CT treatment period and the first week following completion (weeks 4 and 5), and during weeks 9 and 10. During these weeks, participants will continuously wear a wrist actigraph to estimate sleep and light exposure (Philips Actiwatch Spectrum, Philips Respironics, Murrysville, PA, USA) and an accelerometer worn on the trunk to estimate energy expenditure (Philips DirectLife, DL8700/01, Philips, Eindhoven, The Netherlands).

### Compliance

#### CBT-I

Compliance with some aspects of CBT-I, for example sleep restriction and stimulus control, can be monitored using the sleep diary combined with the actigraphic sleep estimates.

#### CT

Compliance with the chronobiological treatments will be monitored by means of questions added to the sleep diaries. Moreover, objective measures of compliance for BL and PA can be derived from ambulatory monitoring of physical activity and light exposure by the Actiwatch Spectrum and DirectLife devices. Participants randomized to BL are instructed to expose the Actiwatch Spectrum to the light when sitting in front of the goLite BLU. Participants assigned to BW receive a digital bath thermometer (type: Lotus, Béaba, Oyonnax Cedex, France) and are asked to register temperature of the bathing water to monitor compliance with the body warming treatment.

### Outcome measures

The primary outcome measure is the change from week 0 to week 5 in subjective sleep efficiency derived from the sleep diary. Secondary subjective sleep estimates include difficulties falling asleep, difficulties maintaining sleep, early morning awakening and non-restorative sleep. A secondary outcome regarding daytime consequences is the average rating of evening diary ratings on eighteen major daytime functioning complaints. Ancillary analyses on persistence of treatment use the same variables obtained in week 10.

Actigraphically estimated objective sleep parameters (sleep efficiency, sleep onset latency, sleep duration, wake after sleep onset, and the average durations of uninterrupted periods of sleep and wakefulness) are secondary outcome measures meant to complement the subjective evaluations. These are calculated from the ActiWatch Spectrum recordings using the Respironics Actiware software (version 5.71.0, Philips Respironics, Murrysville, PA, USA). Given the variability in subjective [88] and objective [89] sleep estimates, they will be assessed for 7 subsequent days at each assessment. Additional questionnaires, assessed in weeks 0, 5 and 10, will provide secondary outcome measures (see the list above). Specifically, the direct and indirect medical costs (TiC-P) will be included.

### Effect moderation by subtype score

Preliminary results of the Latent Class Analysis on comprehensive psychometric data of a large sample of participants of the Netherlands Sleep Registry suggests the existence of four different subtypes of insomnia, each with about equal prevalence. From the extensive number of questionnaires on traits these volunteers filled out, we are presently selecting the minimal subset of questions required to obtain good estimates of the a posteriori probabilities of a participant to belong to each one of the four classes. These items will be included in a Sleep Subtype Survey that will be assessed in all participants of the present RCT. The resulting four Insomnia Subtype Probabilities (ISP) will be included in analyses aimed at evaluating treatment effect moderation by each of the four insomnia subtype probabilities.

### Statistical analysis

#### Data reduction and reporting

The data of qualitative variables (for example, gender, type of treatment, and insomnia subtype) will be presented as incidence rates (number and percent). The data of continuous variables (for example, age, measures derived from sleep diaries, questionnaires and actigraphy) will be presented by measures of central tendency (that is, mean and median) and dispersion (that is, standard deviation and range).

#### Treatment effects and moderation estimates

Mixed effect models will be applied to estimate all time by treatment effects, time by treatment interaction effects and effect moderation by the individual’s insomnia subtype probabilities. For each outcome measure, all effects will be estimated simultaneously in one single linear regression equation. The primary outcome measure of sleep efficiency will be illustrated below. The diary and actigraphy based outcome measures have a two-level hierarchical data-structure: repeated measures of 14 diary inputs *i* across T0 and T1 are nested within subjects *j*. The 14*160 = 2,240 data points can be used to estimate simultaneously all effects ß using the following mixed effect model:

$$ \begin{array}{l}\mathrm{S}{\mathrm{E}}_{\mathrm{ij}}\kern0.5em =\kern0.5em {\mathrm{\ss}}_{0\mathrm{i}\mathrm{j}}\kern0.5em +\kern0.5em {\mathrm{\ss}}_1*{\mathrm{Time}}_{\mathrm{ij}}\kern0.5em +\kern0.5em {\mathrm{\ss}}_2*\mathrm{C}\mathrm{B}\mathrm{T}\hbox{-} {\mathrm{I}}_{\mathrm{j}}\kern0.5em +\kern0.5em {\mathrm{\ss}}_3*\mathrm{B}{\mathrm{L}}_{\mathrm{j}}\kern0.5em +\kern0.5em {\mathrm{\ss}}_4*\mathrm{P}{\mathrm{A}}_{\mathrm{j}}\kern0.5em +\kern0.5em {\mathrm{\ss}}_5*\mathrm{B}{\mathrm{W}}_{\mathrm{j}}\kern0.5em +\kern0.5em {\mathrm{\ss}}_6*\mathrm{IS}{\mathrm{P}}_{\mathrm{j}}\kern0.5em +\kern0.5em {\mathrm{\ss}}_7*\mathrm{T}\mathrm{i}\mathrm{m}{\mathrm{e}}_{\mathrm{ij}}*\mathrm{IS}{\mathrm{P}}_{\mathrm{j}}+\\ {}\kern0.5em {\mathbf{\ss}}_{\mathbf{8}}*\mathbf{C}\mathbf{B}\mathbf{T}\hbox{-} {\mathbf{I}}_{\mathbf{j}}*\mathbf{T}\mathbf{i}\mathbf{m}{\mathbf{e}}_{\mathbf{ij}}\kern0.5em +\kern0.5em {\mathrm{\ss}}_9*\mathrm{C}\mathrm{B}\mathrm{T}\hbox{-} \mathrm{I}*\mathrm{IS}{\mathrm{P}}_{\mathrm{j}}\kern0.5em +\kern0.5em {\mathbf{\ss}}_{\mathbf{10}}*\mathbf{C}\mathbf{B}\mathbf{T}\mathbf{\hbox{-} }{\mathbf{I}}_{\mathbf{j}}*\mathbf{T}\mathbf{i}\mathbf{m}{\mathbf{e}}_{\mathbf{ij}}*\mathbf{I}\mathbf{S}\mathbf{P}+\\ {}\kern0.5em {\mathbf{\ss}}_{\mathbf{11}}*\mathbf{B}{\mathbf{L}}_{\mathbf{j}}*\mathbf{T}\mathbf{i}\mathbf{m}{\mathbf{e}}_{\mathbf{ij}}\kern0.5em +\kern0.5em {\mathrm{\ss}}_{12}*\mathrm{B}\mathrm{L}*\mathrm{IS}{\mathrm{P}}_{\mathrm{j}}\kern0.5em +\kern0.5em {\mathbf{\ss}}_{\mathbf{13}}*\mathbf{B}{\mathbf{L}}_{\mathbf{j}}*\mathbf{T}\mathbf{i}\mathbf{m}{\mathbf{e}}_{\mathbf{ij}}*\mathbf{I}\mathbf{S}{\mathbf{P}}_{\mathbf{j}}+\\ {}\kern0.5em {\mathbf{\ss}}_{\mathbf{14}}*\mathbf{P}{\mathbf{A}}_{\mathbf{j}}*\mathbf{T}\mathbf{i}\mathbf{m}{\mathbf{e}}_{\mathbf{ij}}\kern0.5em +\kern0.5em {\mathrm{\ss}}_{15}*\mathrm{P}\mathrm{A}*\mathrm{IS}{\mathrm{P}}_{\mathrm{j}}\kern0.5em +\kern0.5em {\mathbf{\ss}}_{\mathbf{16}}*\mathbf{P}{\mathbf{A}}_{\mathbf{j}}*\mathbf{T}\mathbf{i}\mathbf{m}{\mathbf{e}}_{\mathbf{ij}}*\mathbf{I}\mathbf{S}{\mathbf{P}}_{\mathbf{j}}+\\ {}\kern0.5em {\mathbf{\ss}}_{\mathbf{17}}*\mathbf{B}{\mathbf{W}}_{\mathbf{j}}*\mathbf{T}\mathbf{i}\mathbf{m}{\mathbf{e}}_{\mathbf{ij}}\kern0.5em +\kern0.5em {\mathrm{\ss}}_{18}*\mathrm{B}\mathrm{W}*\mathrm{IS}{\mathrm{P}}_{\mathrm{j}}\kern0.5em +\kern0.5em {\mathbf{\ss}}_{\mathbf{19}}*\mathbf{B}{\mathbf{W}}_{\mathbf{j}}*\mathbf{T}\mathbf{i}\mathbf{m}{\mathbf{e}}_{\mathbf{ij}}*\mathbf{I}\mathbf{S}{\mathbf{P}}_{\mathbf{j}}+\\ {}\kern0.5em {\mathrm{\ss}}_{20}*\mathrm{C}\mathrm{B}\mathrm{T}\hbox{-} {\mathrm{I}}_{\mathrm{j}}*\mathrm{B}{\mathrm{L}}_{\mathrm{j}}\kern0.5em +\kern0.5em {\mathbf{\ss}}_{\mathbf{21}}*\mathbf{C}\mathbf{B}\mathbf{T}\mathbf{\hbox{-} }{\mathbf{I}}_{\mathbf{j}}*\mathbf{B}{\mathbf{L}}_{\mathbf{j}}*\mathbf{T}\mathbf{i}\mathbf{m}{\mathbf{e}}_{\mathbf{ij}}\kern0.5em +\kern0.5em {\mathrm{\ss}}_{22}*\mathrm{C}\mathrm{B}\mathrm{T}\hbox{-} {\mathrm{I}}_{\mathrm{j}}*\mathrm{B}{\mathrm{L}}_{\mathrm{j}}*\mathrm{IS}{\mathrm{P}}_{\mathrm{j}}\kern0.5em +\kern0.5em {\mathbf{\ss}}_{\mathbf{23}}*\mathbf{C}\mathbf{B}\mathbf{T}\hbox{-} \\ {}\kern0.5em {\mathbf{I}}_{\mathbf{j}}*\mathbf{B}{\mathbf{L}}_{\mathbf{j}}*\mathbf{T}\mathbf{i}\mathbf{m}{\mathbf{e}}_{\mathbf{ij}}*\mathbf{I}\mathbf{S}\mathbf{P}+\\ {}\kern0.5em {\mathrm{\ss}}_{24}*\mathrm{C}\mathrm{B}\mathrm{T}\hbox{-} {\mathrm{I}}_{\mathrm{j}}*\mathrm{P}{\mathrm{A}}_{\mathrm{j}}\kern0.5em +\kern0.5em {\mathbf{\ss}}_{\mathbf{25}}*\mathbf{C}\mathbf{B}\mathbf{T}\mathbf{\hbox{-} }{\mathbf{I}}_{\mathbf{j}}*\mathbf{P}{\mathbf{A}}_{\mathbf{j}}*\mathbf{T}\mathbf{i}\mathbf{m}{\mathbf{e}}_{\mathbf{ij}}\kern0.5em +\kern0.5em {\mathrm{\ss}}_{26}*\mathrm{C}\mathrm{B}\mathrm{T}\hbox{-} {\mathrm{I}}_{\mathrm{j}}*\mathrm{P}{\mathrm{A}}_{\mathrm{j}}*\mathrm{IS}{\mathrm{P}}_{\mathrm{j}}\kern0.5em +\kern0.5em {\mathbf{\ss}}_{\mathbf{27}}*\mathbf{C}\mathbf{B}\mathbf{T}\mathbf{\hbox{-}}\\ {}\kern0.5em {\mathbf{I}}_{\mathbf{j}}*\mathbf{P}{\mathbf{A}}_{\mathbf{j}}*\mathbf{T}\mathbf{i}\mathbf{m}{\mathbf{e}}_{\mathbf{ij}}*\mathbf{I}\mathbf{S}\mathbf{P}\mathbf{j}+\\ {}\kern0.5em {\mathrm{\ss}}_{28}*\mathrm{C}\mathrm{B}\mathrm{T}\hbox{-} {\mathrm{I}}_{\mathrm{j}}*\mathrm{B}{\mathrm{W}}_{\mathrm{j}}\kern0.5em +\kern0.5em {\mathbf{\ss}}_{\mathbf{29}}*\mathbf{C}\mathbf{B}\mathbf{T}\mathbf{\hbox{-} }{\mathbf{I}}_{\mathbf{j}}*\mathbf{B}{\mathbf{W}}_{\mathbf{j}}*\mathbf{T}\mathbf{i}\mathbf{m}{\mathbf{e}}_{\mathbf{ij}}\kern0.5em +{\mathrm{\ss}}_{30}*\mathrm{C}\mathrm{B}\mathrm{T}\hbox{-} {\mathrm{I}}_{\mathrm{j}}*\mathrm{B}{\mathrm{W}}_{\mathrm{j}}*\mathrm{IS}{\mathrm{P}}_{\mathrm{j}}\kern0.5em +\kern0.5em {\mathbf{\ss}}_{\mathbf{31}}*\mathbf{C}\mathbf{B}\mathbf{T}\mathbf{\hbox{-}}\\ {}\kern0.5em {\mathbf{I}}_{\mathbf{j}}*\mathbf{B}{\mathbf{W}}_{\mathbf{j}}*\mathbf{T}\mathbf{i}\mathbf{m}{\mathbf{e}}_{\mathbf{ij}}*\mathbf{I}\mathbf{S}{\mathbf{P}}_{\mathbf{j}}\end{array} $$

In this regression equation, SE = sleep efficiency; ß_0ij_ = intercept; time denotes the post intervention (T1) versus baseline (T0) assessment week; CBT = cognitive behavior therapy assignment; BL = bright light assignment; PA = physical activity assignment; BW = body warming assignment; ISP = insomnia subtype probability; and ß_1_ to ß_31_ denote effect estimates. The effects and terms of interest are shown in bold font. For example, line 2 shows in bold font the overall treatment by time interaction effect **ß**_**8**_ of CBT-I versus waitlist and the moderation of this effect **ß**_**10**_ by one’s insomnia subtype probability. Lines 3 to 5 show the corresponding effect estimates for BL, PA and BW interventions, and lines 6 to 8 the for their interaction with CBT-I. For clarity, the regression equation shows only one of the four expected ISPs. Wald tests will be used to evaluate the significance of the effect estimates. Statistical significance is thresholded at p = 0.05. The simultaneous estimation of all effects of interest in a single regression model compensates for multiple comparisons. Mixed effect model analyses will be implemented in MLwiN (version 2.02, Centre for Multilevel Modelling, University of Bristol, Bristol, UK.) and (R: A language and evironment for statistical computing, R Foundation for Statistical Computing, Vienna, Austria.).

### Power and sample size

#### Statistical power estimates

The statistical power of mixed effect regression models with seven repeated measures (diaries) both before and after intervention have been calculated according to the equations given by Twisk, page 280 [90], similar to those calculations made in a previous repeated measures RCT that we published [91]. Statistical power calculations on repeated measures require an estimate of the intraclass correlation coefficient (ICC, that is, within-subject). We estimated the ICC for the primary outcome measure of subjective sleep efficiency from the 9-day sleep diaries [67] that we recently assessed through the internet for 27 people suffering from insomnia according to an Insomnia Severity Index ≥10 [92]. In agreement with the robust observation that night-by-night sleep variability is an intrinsic part of the insomnia symptomatology (reviewed in [88]), we found only a moderate ICC of 0.27, supporting the use of repeated assessment of the primary outcome measure to increase statistical power of the design.

Given this moderate ICC of 0.27, seven pre-assessments at T0, and seven post-assessments at T1, the 160 completing participants provide a power 1-beta of 0.80 to allow for the detection, at a significance of alpha = 0.05, of even small to medium main effects of CBT-I (minimal detectable difference d = 0.27 standard deviations), of each of the three CTs (d = 0.31) and of their interaction with CBT-I (d = 0.41). The minimally detectable difference in the time by treatment interaction between any two types of CT is small to medium as well (d = 0.38).

There is unfortunately no readily available methodology to estimate the minimally detectable moderation effects of continuous variables (insomnia subtype probabilities) on a time by treatment interaction effect on a continuous outcome variable (sleep efficiency). In order to obtain a conservative estimate of minimally detectable moderation effects, we can dichotomize the continuous insomnia subtype probability variable by means of a median split, effectively generating two equal groups. The design then becomes one of three factors, each with two levels: time (T1 versus T0), treatment group (Treated versus Untreated on the specific intervention) and insomnia subtype (high versus low probability) and provides the ratio of available data in the subgroup or interest relative to the other available data, to be used once more in the equations given by Twisk, page 280 [90]. According to this conservative approach of mapping the continuous insomnia subtype probabilities onto a dichotomous variable, the 160 completing participants provide a power 1-beta of 0.80 to allow for the detection, at a significance of alpha = 0.05: of a small to medium moderation effects of insomnia subtype (1) on the time by CBT-I treatment interaction (d = 0.31); (2) on the time by each CT treatment interaction (d = 0.41); and (3) on the difference in the time by treatment interaction between any two types of CT (d = 0.44). The smallest detectable moderation effect of insomnia subtype on the time by combined CBT-I and CT interaction is medium sized (d = 0.55). We follow the convention on effect size given by Cohen [93], who suggested consideration of an effect size d of about 0.2 as “small” and an effect size of about 0.5 as “medium.” With an estimated dropout of 10 %, we will recruit 180 volunteers. The expected 160 completers thus allow for the detection of small to medium effect sizes.

## Discussion

The ultimate goal of this study is to develop a personalized treatment protocol for insomnia disorder. Primary caregivers could use this protocol to decide on the primary choice treatment for each individual patient. Assessment of insomnia subtype probabilities using the Sleep Subtype Survey results in four ISPs, which can be used to determine the expected treatment outcome of each of four treatments, their combinations and their simultaneous versus sequential application for the individual patient.

Since all treatments used in this study are noninvasive, easily applicable at home and require only the use of a computer and Internet (CBT-I) and/or a readily available therapy light (BL), the implementation of treatments is straightforward, low-cost and scalable.

The current study addresses multiple opportunities to optimize and personalize treatment of insomnia disorder. First, no prior study has evaluated the relative effectiveness of CBT-I, the three different forms of CT, and their simultaneous or sequential combinations. Second, the moderation of outcome by insomnia subtype probability follows the principles of personalized and stratified medicine. Treatment effects with subtype-specificity may come to light that would have remained hidden in the unrecognized heterogeneity of the samples.

## Trial status

Recruitment started in October 2013 and is ongoing. In January 2014, the first participants enrolled. Currently, 60 participants have completed the protocol.

## Abbreviations

APS: arousal predisposition scale
ARSQ: Amsterdam resting state questionnaire
ASRS: ADHD self-report scale
BL: bright light treatment
BW: body warming treatment
CBT: cognitive behavioral therapy
CBT-I: cognitive behavioral therapy for insomnia
CSD: consensus sleep diary
CT: chronobiological treatment
DI: deactivated ionizer
DBAS: dysfunctional beliefs and attitudes towards sleep
DSISD: Duke’s Structured Interview for Sleep Disorders
DSM-V: diagnostic and statistical manual of mental disorders, 5th edition
EMA: early morning awakening
GSES: Glasgow sleep efficiency scale
HADS: hospital anxiety and depression scale
HAS: hyperarousal scale
HDNI: high density negative ionization
ICC: intraclass correlation coefficient
ICSD-2: international classification of sleep disorders, 2nd edition
ID: insomnia disorder
ISI: insomnia severity index
ISP: insomnia subtype probability
OR: odds ratio
PA: physical activity treatment
PANAS: positive affect, negative affect scale
PSAS: pre-sleep arousal scale
SCN: suprachiasmatic nucleus
SE: sleep efficiency
SF-36: short form 36
SLOC: sleep locus of control
SOL: sleep onset latency
SSES: sleep self-efficacy scale
TEPS: temporal experience of pleasure scale
TEPS: temporal experience of pleasure scale
TiC-P: Trimbos/iMTA questionnaire for costs associated with psychiatric illness
WASO: wake after sleep onset
